# Cathepsin-L Secreted by High-Quality Bovine Embryos Exerts an Embryotrophic Effect In Vitro

**DOI:** 10.3390/ijms24076563

**Published:** 2023-03-31

**Authors:** Annelies Raes, Eline Wydooghe, Krishna Chaitanya Pavani, Osvaldo Bogado Pascottini, Katleen Van Steendam, Maarten Dhaenens, Annekatrien Boel, Sonia Heras, Björn Heindryckx, Luc Peelman, Dieter Deforce, Filip Van Nieuwerburgh, Geert Opsomer, Ann Van Soom, Katrien Smits

**Affiliations:** 1 Department of Internal Medicine, Reproduction and Population Medicine, Faculty of Veterinary Medicine, Ghent University, B-9820 Merelbeke, Belgium; 2 Laboratory for Pharmaceutical Biotechnology, Faculty of Pharmaceutical Science, Ghent University, B-9000 Ghent, Belgium; 3 Ghent-Fertility and Stem Cell Team, Department for Reproductive Medicine, University Hospital, Ghent University, B-9000 Ghent, Belgium; 4 Department of Physiology, Faculty of Veterinary Medicine, University of Murcia, 30071 Murcia, Spain; 5 Department of Veterinary and Biosciences, Faculty of Veterinary Medicine, Ghent University, B-9820 Merelbeke, Belgium

**Keywords:** in vitro culture, embryonic development, embryotrophins

## Abstract

While human in vitro embryo production is generally performed individually, animal models have shown that culturing embryos in groups improves blastocyst yield and quality. Paracrine embryotrophins could be responsible for this improved embryo development, but their identity remains largely unknown. We hypothesize that supplementation of embryotrophic proteins to a culture medium could be the key to improve individual embryo production. In this study, proteomics screening of culture media conditioned by bovine embryos revealed cathepsin-L as being secreted by both excellent- and good-quality embryos, while being absent in the medium conditioned by poor-quality embryos. The embryotrophic role of cathepsin-L was explored in vitro, whereby bovine zygotes were cultured individually for 8 days with or without cathepsin-L. Preliminary dose–response experiments pointed out 100 ng/mL as the optimal concentration of cathepsin-L in embryo culture medium. Supplementation of cathepsin-L to individual culture systems significantly improved blastocyst development and quality in terms of blastocoel formation at day 7, and the hatching ratio and apoptotic cell ratio at day 8, compared to the control. Taken together, cathepsin-L acts as an important embryotrophin by increasing embryo quality, and regulating blastulation and hatching in bovine in vitro embryo production.

## 1. Introduction

In vitro production of mammalian embryos has advanced significantly over the last decades, resulting in the use of assisted reproduction as infertility treatment for humans, domesticated, and wild animal species [1,2,3,4]. Further attempts to improve embryo culture systems have been mainly focused on enhancing media formulations [5,6]. Interestingly, animal studies have shown that culturing embryos in groups, as opposed to individual culture, improves blastocyst cell number and viability as far as three decades ago [7,8,9,10,11]. The positive effect of group culture has been attributed to the secretion of auto- and paracrine factors [10]. These factors serve as signaling molecules produced and released by embryos, and act upon the embryo itself or the neighboring embryos [12,13,14,15]. A wide range of biochemical messengers, including proteins, lipids, neurotransmitters, saccharides, and nucleic acids, can be exchanged among embryos cultured in groups and may act as potential embryotrophic mediators [16]. The embryo secretome, i.e., proteins secreted within the culture medium by preimplantation embryos, was explored in human [17,18,19,20,21], murine [22,23], and equine [24] studies.

Secretome analysis of in-vitro-produced embryos allows researchers to explore important biomarkers that characterize viable embryos with adequate implantation potential [25,26]. Most embryologists select good-quality embryos based on morphological evaluation by standard microscopy, but this method is strongly influenced by inter-observer variations and has low levels of accuracy [27]. Secretome analysis could serve as an alternative non-invasive tool for embryo selection in a more objective manner with improved reproducibility [28], and it is becoming more commonly used as a non-invasive tool to predict successful embryo implantation [25,29,30,31,32,33,34] or to select chromosomally stable embryos for transfer in human fertility clinics [35]. Accordingly, the identification of the secretome in animal models could help researchers provide novel insights into early embryonic development and collaborate to unravel key aspects of the complex embryo-maternal dialogue. Several studies focused on the secretion of immunological factors by human and murine embryos, as they play a major role in the embryo-maternal dialogue by facilitating immune-related implantation events (for review, see Bahrami-Asl [25]). Granulocyte–macrophage colony-stimulating factor, a pertinent regulator for the immune response during implantation, was detected in the secretome of both human [36] and murine [37] embryos, and was considered a potential marker of viable blastocysts [36,37,38]. Katz-Jaffe and colleagues [31] reported similar protein profiles between the human and murine secretome, and proposed the regulatory protein ubiquitin as a candidate biomarker for blastocyst development in both species [31]. Other candidate biomarkers of embryo viability that were identified in the human secretome are human chorionic gonadotropin [39], apolipoprotein A1 [40], and lipocalin-1 [35], amongst others, whereas acrogranin, a protein that regulates epithelial cell growth, was identified as embryotrophic mediator in the secretome of murine embryos [41]. Calle and colleagues [42] explored the secretome of bovine trophoblast cells and its effect on maternal mesenchymal stem cell lines. However, a non-target approach to identify the components of the embryo secretome was not yet performed in cattle. This is partly attributed to technical limitations in the detection of the low-abundant proteins of interest compared to the high abundance of albumin, which is a common supplement of the embryo culture media. The use of an albumin-free medium can increase the number of identified proteins, as shown in a study on the secretome of murine embryos [23]. Additionally, the substantial number of arrested and apoptotic cells in in-vitro-produced embryos should be considered [43,44,45], since their cell membranes may be damaged, which can cause a passive leakage of proteins to the culture medium [46]. Since this could mask actively secreted autocrine factors, individual culture is required, and the ratio of membrane-damaged cells (MDCs) should be taken into consideration as a quality parameter when secretome analysis is performed. 

Cathepsin-L, a member of the cathepsin family, was pointed out as one of the main secreted proteins by equine embryos [24]. Cathepsins are cysteine proteases that can be found in lysosomes, cytosol, and the extracellular matrix, are mainly involved in protein catabolism [47]. As such, the role of cathepsin-L proteolytic activity in the establishment of the embryo-maternal dialogue was studied in different species [48,49,50,51,52,53] and reviewed by Spencer and colleagues [54]. Moreover, the contribution of cathepsin-L to cell differentiation was highlighted in several publications [55,56,57].

The objective of this study was to identify proteins actively secreted by in-vitro-produced bovine embryos and to further evaluate their potential role as an embryotrophic factor in the in vitro culture system. Spent culture medium of individually cultured embryos was categorized based on embryo quality, and a screening of present peptides was performed by liquid chromatography and tandem mass spectrometry (MS). The resulting protein of interest, cathepsin-L, which was secreted by embryos of excellent and good quality, was subsequently supplemented to the embryo’s culture medium, where it stimulated embryonic development.

## 2. Results

### 2.1. Cathepsin-L Is Secreted by Embryos of Excellent and Good Quality

#### 2.1.1. Classification of Embryo Quality: Membrane-Damaged Cell Ratio

**Figure 1:**
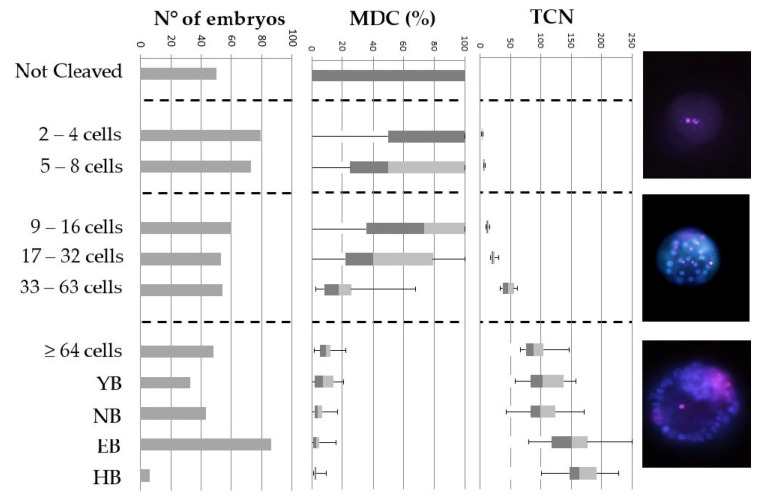
Prevalence of membrane-damaged cells (MDCs) and total cell number (TCN) in bovine embryos, at day 8 post-fertilization. Embryos were cultured individually in droplets of 20 µL albumin-free culture medium and at day 8, a morphological evaluation of the blastocyst stage was performed using a stereomicroscope. Subsequently, all non-fixed viable embryos were subjected to Hoechst/propidium iodide staining to assess the TCN and the percentage of MDC. The boxplots show the median values (transition dark grey–light grey), 25th and 75th percentile (boxes), and 5th and 95th percentile (whiskers). All embryos (n = 606) were collected from four replicates, the number of embryos belonging to a developmental stage (YB = young blastocyst; NB = normal blastocyst; EB = expanded blastocyst; HB = hatching or hatched blastocyst) is represented in the figure.

The quality of 606 embryos, cultured individually in an albumin-free medium, was determined at day 8 post-fertilization, based on the total cell number (TCN) and MDC ratio (Figure 1). Twenty-six embryos had a TCN of at least 64 cells expressing no membrane damage. These embryos were defined as excellent-quality embryos. Ninety embryos were defined as good-quality embryos, as they had a TCN of 64 cells or more, in combination with a limited MDC ratio (0.01 to 5%). Embryos that did not reach the 64-cell stage and had an MDC ratio of more than 90% were categorized as poor-quality embryos (n = 138). The remaining embryos had a sufficient TCN (≥64 cells), but a high MDC ratio (>5%; n = 91) or did not reach the 64-cell stage, although having a relatively low MDC (<90%; n = 261). The latter embryos were not used for further processing. 

#### 2.1.2. Protein Identification by Mass Spectrometric Analysis

**Figure 2:**
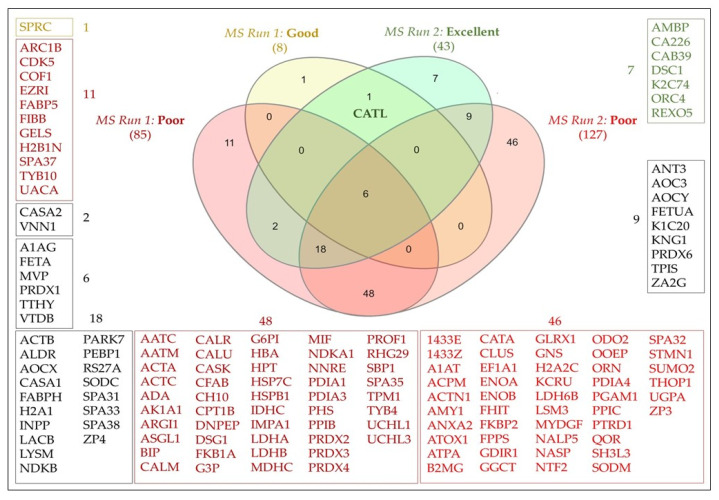
Venn diagram depicting all protein accessions identified in the compared media after subtraction of the proteins in the blank medium. Compared media: ‘MS Run 1: Good and Poor’: pooled medium conditioned by good- and poor-quality embryos, respectively; ‘MS Run 2: Excellent and Poor’: pooled medium conditioned by excellent- and poor-quality embryos, respectively. Numbers from the Venn diagram are depicted with their respective Uniprot accession numbers in the periphery. Protein descriptions can be found in Supplementary Tables S1 and S2. Cathepsin-L (CATL) was the only protein exclusively identified in ‘MS Run 1: Good’ and ‘MS Run 2: Excellent’.

The secretome of embryos was examined performing tandem MS analysis on embryo-conditioned medium and unconditioned (blank) culture medium. In the blank medium, peptides from insulin, serum albumin and transferrin were identified, alongside common laboratory contaminants, such as keratins and trypsin. After subtraction of these peptides, a total of 149 proteins remained in the medium conditioned by embryos. MS Run 1 identified eight proteins in the medium conditioned by good-quality embryos, and 85 proteins in the corresponding medium conditioned by poor-quality embryos (Figure 2, Supplementary Table S1). In MS Run 2, 43 proteins were detected in the conditioned medium of excellent-quality embryos, while 127 proteins were detected in the medium conditioned by corresponding poor-quality embryos (Figure 2, Supplementary Table S2).

Seven proteins were secreted exclusively by excellent-quality embryos (AMBP, CA226, CAB39, DSC1, K2C74, ORC4 and REXO5), one protein was unique to the good-quality group (SPRC) and 48 proteins were solely secreted by poor-quality embryos in both MS runs (Figure 2). Only one protein was present in the embryo conditioned medium of both good- and excellent-quality embryos, but was not identified in the pool of medium conditioned by poor-quality embryos nor in the blank: cathepsin-L (CATL_BOVIN). As cathepsin-L has previously been involved in early embryonic development [53,55], its potential role as an embryotrophic marker was examined. Of note, the selection of proteins that are unique to embryos of good and excellent quality could also be of potential interest for future studies.

### 2.2. In Vitro Confirmation of the Embryotrophic Effect of Cathepsin-L

#### 2.2.1. Cathepsin-L Advances Blastulation and Hatching in Individual Embryo Culture

Cathepsin-L supplementation (100 ng/mL; concentration determined by preliminary dose-response experiments, Supplementary Table S3) in the individual culture system resulted in a higher blastocyst rate at day 7 post-fertilization (27.6 ± 1.83%) compared with the control medium in individual culture (20.9 ± 1.64%; *p* = 0.0196). The hatching rate of cathepsin-L-derived blastocysts was significantly higher compared to the control in the individual culture (19.9 ± 2.67% versus 7.67 ± 1.81%, respectively, *p* = 0.0071) and was similar to the control in the group culture (17.1 ± 1.81%; *p* = 0.6976) at day 8 post-fertilization (Table 1). 

#### 2.2.2. Differential Apoptotic Staining Reveals Decreased Apoptotic Cell Ratio after Cathepsin-L Supplementation

Cathepsin-L supplementation to the culture medium yielded blastocysts with fewer apoptotic cells compared to both control groups (4 ± 2 versus 6 ± 4 and 5 ± 4 for cathepsin, control group and control individual, respectively; *p* ≤ 0.0162). In line with this finding, a lower apoptotic cell ratio was detected in the embryos treated with cathepsin-L (3.14 ± 2.58) compared to the controls (4.92 ± 4.00 and 4.35 ± 3.32 for group and individual control, respectively; *p* ≤ 0.0459) (Table 2). However, no significant differences were observed in the total cell number and inner cell mass ratio between the different treatment groups (*p* ≥ 0.913 Table 2). 

## 3. Discussion

To study the potential role of embryotrophins in individual culture systems for in vitro embryo production, a screening of the secretome of preimplantation bovine embryos was performed. This study identified cathepsin-L secretion by in-vitro-cultured embryos of excellent and good quality only. The embryotrophic effect of cathepsin-L was demonstrated by increased blastulation and hatching rates, and a reduced apoptotic cell ratio when cathepsin-L was supplemented to the medium of individual embryo cultures. Therefore, (1) the presence of cathepsin-L in embryo-conditioned culture medium could be indicative of good embryo quality, whereby its role as potential biomarker for non-invasive selection of transferable embryos should be further explored and (2) supplementation of cathepsin-L to the culture medium may ameliorate embryogenesis in individual culture systems. 

Tandem MS screening of embryo-conditioned culture medium resulted in the identification of 149 embryo-secreted proteins associated with embryo quality, by optimizing individual culture of embryos in albumin-free medium and live staining. Embryos were cultured individually so that neither embryonic development nor the associated culture medium was influenced by neighboring embryos and a direct link of the conditioned medium with the corresponding embryo was permitted. Consequently, the collected medium could be distinguished between different embryo quality groups, which were divided based on the embryo TCN and MDC ratio. Membrane damage allows for a passive release of intracellular proteins into the medium, which could erroneously be accounted as part of the secretome. By using live staining with Hoechst and propidium iodide, it was confirmed that the identified proteins in the culture medium conditioned by good- and excellent-quality embryos were actively secreted employing the constitutive secretory pathway or through extracellular vesicles [16,58], and are not passively released by membrane-damaged blastomeres. Only a small percentage of the embryos showed no MDCs at all, even if the embryos had at least 64 cells at day 8 post-fertilization. Almost half of the arrested embryos with 64 cells or less at day 8 had an MDC ratio of more than 50%, indicating that poor-quality embryos consist predominantly of MDCs and, indeed, more proteins were identified in the medium conditioned by poor-quality embryos. Forty-eight proteins were shared between both pools of the culture media conditioned by poor-quality embryos. However, due to the high MDC ratio of poor-quality embryos and the potential passive leakage of proteins into the extracellular environment, these proteins cannot be assigned with certainty to the poor-quality embryo secretome. This could explain why several proteins that are vital for successful embryo development were detected in the medium conditioned by poor-quality embryos in the present study. Calmodulin, for example, was present in the medium of poor-quality embryos, while being linked to successful blastocoel formation in mouse embryos [59]. Furthermore, IDHC [60], PRDX2 [60,61] and glucose-6-phosphatase isomerase [62] were highlighted as predictors of embryo implantation, while being detected solely in the medium conditioned by poor-quality embryos. On the other hand, LDHA1, a protein responsible for lactate synthesis, was also unique to the poor-quality medium in this study and was additionally linked to human infertility, since LDHA1 concentrations were higher in decidual trophoblast of women with recurrent miscarriage compared to women with normal pregnancies [63].

To obtain a reliable identification of low-abundant proteins in the secretome, an albumin-free medium was used. To do so, polyvinylpyrrolidone (PVP) served as albumin substitute, since high-abundant proteins, such as albumin, could mask the presence of the low-abundant proteins of interest [23]. Identification of the proteins secreted by bovine embryos was accomplished, despite the presence of albumin remnants derived from HEPES–TALP (Tyrode’s albumin lactate pyruvate) medium used in the in vitro fertilization procedure prior to the embryo culture. Further reduction of albumin concentration by using albumin-free HEPES–TALP medium was not attempted, since a previous study showed that the replacement of bovine serum albumin (BSA) by PVP could adversely affect embryo development [64]. As such, a trade-off between embryo development on one side and a reduction of analytical interference on the other side was made. Transcriptome analysis data revealed the presence of the cathepsin-L gene in bovine in-vivo-derived pre-implantation embryos from the 8-cell stage, up to the blastocyst stage, with the highest expression during the blastocyst stage (although this was not stage-specific) [65]. Another study reported the presence of procathepsin-L in in-vitro- and in-vivo-derived bovine embryos, starting from the 8-cell and 4-cell stages, respectively. In both cases, expression of procathepsin-L peaked at the blastocyst stage and no significant differences were obtained between in vitro and in vivo embryos [66]. Likewise, studies in human and marmoset embryos reported the expression of cathepsin-L starting from the zygote, up to the blastocyst stage, with highest expression at the blastocyst stage [67,68]. In mouse embryos, cathepsin-L expression was detected from the zygote, until the blastocyst stage, although upregulation was noticed during the 2-to-8-cell stages [69]. Overall, these results suggest that the action of cathepsin-L is required after embryonic genome activation in all considered species. Interestingly, Ushizawa and colleagues [70] reported upregulation of the cathepsin-L gene between bovine embryos collected at day 14 and day 21 of gestation, indicating that the relevance of cathepsin-L increases around the time frame of implantation [70].

In this study, secretome analysis of an embryo-conditioned medium identified cathepsin-L (CATL_BOVIN) as the only protein which was present in the medium conditioned by embryos of both excellent- and good-quality, and absent in the medium conditioned by poor-quality embryos, and in the blank medium. A review by Dhaenens and colleagues [56] pointed out that cathepsin-L is involved in early stem cell differentiation in mouse, where it induces a histone clipping event [56]. As cathepsin-L has previously been involved in early embryonic development [53,55] and has been identified in the secretome of equine blastocysts [24], its potential role as an embryotrophic factor was pursued. The biological relevance of this protein for preimplantation embryo development was examined by supplementing 100 ng/mL human Cathepsin-L (77% homology with CATL_BOVIN and 100% homology for active sites, UNIPROT) to the culture medium of individually cultured bovine embryos. 

Cathepsin-L supplementation (100 ng/mL) to the culture medium in an individual system significantly enhanced blastulation with regard to more embryos reaching the blastocyst stage (day 7 post-fertilization) and improved hatching rates (day 8 post-fertilization) compared to the control. This finding is in line with experiments in sea urchins, where the addition of a cathepsin-L inhibitor to the culture medium during early cleavage cell cycles affected chromosome decondensation and blocked the S-phase of the subsequent cell cycle, and as such, impaired early preimplantation embryo development [71]. Similar results were reported in cancer research, where cathepsin-L is well known to be involved in rapid cell proliferation [72,73]. Furthermore, it has been demonstrated that many cancer cells secrete cathepsin-L into the extracellular milieu, where it plays a role in tumor invasion and metastasis [74]. Although tumor invasion is a more destructive process, many similarities do exist between tumor invasion and embryo implantation, such as a similar molecular regulation of angiogenesis or an equivalent molecular breakdown of the extracellular matrix in species with invasive implantation (e.g., human, mouse) [75,76]. Moreover, the quality of obtained blastocysts was improved after cathepsin-L supplementation in terms of fewer apoptotic cells and a lower apoptotic cell ratio compared to the control group. This is in accordance with the work of Zheng and colleagues [77], who described cathepsin-L as a pro-survival protease, since it was crucial in regulating the levels of apoptosis-associated proteases, cathepsin-D and caspase-3. In addition, during zona hatching, the significant role of cathepsin-L was affirmed in the present study, as cathepsin-L supplementation resulted in more hatched embryos compared to the control in individual culture. The relevance of cathepsin-L during hatching was previously shown in the golden hamster, where it amplified upon the lysis of the zona pellucida and subsequent blastocyst hatching [53]. Moreover, in murine blastocysts it was demonstrated that hatching embryos express cathepsin-L mainly in their trophoblast cells, and dormant embryos had an aggregated cathepsin-L expression in the inner cell mass [78]. Besides facilitating early embryonic development, the proteolytic activity of cathepsin-L contributes to the process of implantation, since it is involved in the interaction between maternal decidua and fetal trophoblast cells, both in species with a non-invasive placenta, such as pigs and sheep [48,49], and in species with an invasive placenta, such as mice [41,42,50,51] and humans [52].

In the present manuscript, secretome analysis linked to the quality of individually cultured embryos resulted in the identification of seven proteins (AMBP, CA226, CAB39, DSC1, K2C74, ORC4 and REXO5) that were unique to excellent-quality embryos. These proteins are candidates for future research considering non-invasive embryo selection. For example, alpha-2 macroglobulin (AMBP) was already proposed as a potential urinary biomarker for early pregnancy in cattle by Rawat and colleagues [79]. Ubiquitin [31], peroxiredoxin-1 [22] and vitamin-D-binding protein [80] were previously detected in the secretome of human or murine preimplantation embryos, and were proposed as mediators for reproductive health [61,81,82]. In the present study, these proteins were detected in the secretome of good- and/or excellent-quality bovine embryos, but also in the medium conditioned by poor-quality embryos. Their presence in the good/excellent-quality embryo secretome confirms earlier results, and thereby suggests the potential relevance of these proteins for successful bovine embryogenesis.

Apart from cathepsin-L, only one protein was encountered in the secretome of good-quality embryos that belonged solely to this group: SPRC_BOVIN (SPARC). Notably, SPARC, a protein associated with cellular remodeling and proliferation, was detected in the conditioned medium of excellent-quality embryos as well, although its expectancy value here was beyond the identity threshold (0.056 instead of 1). Since previous studies pointed out that SPARC is involved in the embryo-maternal crosstalk during placentation [83,84,85], its properties could be explored in future studies. Furthermore, future in vivo studies that implement the transfer of in-vitro-produced embryos to a recipient could (1) confirm the potential role of cathepsin-L in conditioned culture medium as a biomarker for high-quality transferable embryos, and (2) determine the potential effect of cathepsin-L supplementation during in vitro embryo culture on implantation and placentation.

## 4. Materials and Methods

**Figure 3:**
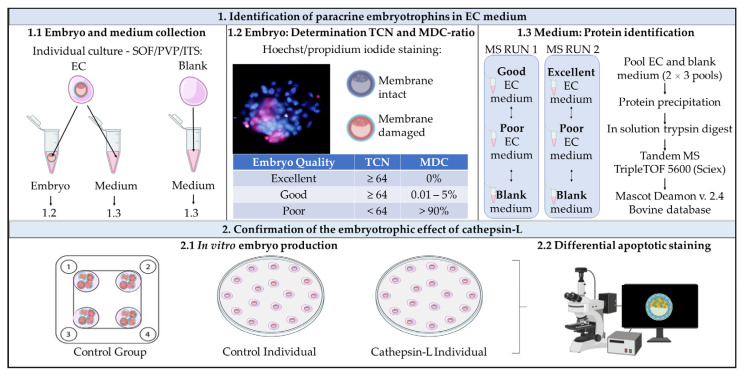
Visual representation of the research described in the present manuscript. 1. Identification of paracrine embryotrophins in embryo-conditioned medium: (1.1) Embryo and medium collection: individual culture of bovine embryos in albumin-free SOF/PVP/ITS medium. Droplets of SOF/PVP/ITS medium without embryo were incubated under the same conditions and served as a blank control. At day 8 post-fertilization, each embryo, droplets of EC medium and droplets of blank medium were collected separately. (1.2) Determination of TCN and MDC ratio: all embryos were subjected to a Hoechst/propidium iodide staining to differentiate between membrane intact and MDCs. Based on the results, embryo quality was categorized as ‘excellent’, ‘good’ or ‘poor’ quality in this context. (1.3) Protein identification: EC medium of good-quality embryos was pooled and compared to pooled EC medium of an equivalent number of poor-quality embryos and an equivalent volume of blank droplets in MS run 1. Under similar conditions, pooled EC medium of excellent-quality embryos, pooled EC medium of poor-quality embryos and blank medium were compared in MS run 2. 2. Confirmation of the embryotrophic effect of cathepsin-L. (2.1) In vitro embryo production: individual culture of bovine embryos in the control medium was compared to individual culture in cathepsin-L-supplemented medium (100 ng/mL). Embryos cultured in a group in the control medium were included as an extra control group. (2.2) Differential apoptotic staining: blastocysts collected at day 8 post-fertilization from exp 2.1 were subjected to differential apoptotic staining. Abbreviations: EC, embryo conditioned; ITS, insulin transferrin selenium; MDC, membrane-damaged cell; MS, mass spectrometry; PVP, polyvinylpyrrolidone; SOF, synthetic oviductal fluid; TCN, total cell number.

A schematic overview of the workflow of this manuscript is presented in Figure 3. 

### 4.1. Media and Reagents

Tissue culture medium (TCM)-199, gentamycin and phosphate-buffered saline (PBS) were purchased from Gibco^TM^, Life Technologies Europe (Ghent, Belgium). All other components, not otherwise listed, were obtained from Sigma-Aldrich (Overijse, Belgium). All media were filter-sterilized before use (0.22 µm Whatman Puradisc filters, Cytiva, Maidstone, UK).

### 4.2. Identification of Autocrine Embryotropins in Embryo-Conditioned Culture Medium

#### 4.2.1. Collection of Embryo-Conditioned Media

Bovine embryos (n = 612, over 4 replicates) were produced by previously described routine in vitro methods [86]. In brief, bovine ovaries were collected at the local slaughterhouse and processed within 2 h. Ovaries were washed three times in warm PBS supplemented with kanamycin (25 mg/mL). Cumulus–oocyte complexes were aspirated from antral follicles (4–8 mm diameter) using an 18 G needle attached to a syringe. Oocytes with a uniformly granulated ooplasm, intact zona pellucida, and surrounded by at least five layers of compact cumulus cells were selected for in vitro maturation and placed in groups of 60 in 4-well dishes filled with 500 μL modified bicarbonate-buffered TCM-199, supplemented with 20 ng/mL epidermal growth factor and 50 µg/mL gentamicin for 22 h at 38.5 °C in 5% CO_2_ in air. Matured oocytes were washed in 500 μL IVF–TALP medium and incubated in 4-well dishes with Percoll (GE Healthcare, Cytiva, Maidstone, UK)-selected frozen–thawed sperm (1 × 10^6^ sp/mL) in IVF–TALP with heparin (25 μg/mL), for 21 h at 38.5 °C in 5% CO_2_ in air. Next, excessive sperm and cumulus cells were removed by vortexing and presumed zygotes were transferred individually to droplets of 20 µL of synthetic oviduct fluid supplemented with essential and non-essential amino acids (SOFaa; Life Technologies Europe, Ghent, Belgium), ITS (5 µg/mL insulin + 5 µg/mL transferrin + 5 ng/mL selenium) and 0.1 mg/mL PVP (SOF/ITS/PVP). The individual droplets were covered with paraffin oil (SAGE, CooperSurgical, Malov, Denmark) and incubated at 38.5 °C in 5% CO_2_, 5% O_2_ and 90% N_2_. An equivalent volume of blank SOF/ITS/PVP medium (i.e., not conditioned by embryos) was incubated in the same conditions. At day 8 post-fertilization, morphological evaluation of the blastocyst stage was performed using an inverted stereomicroscope and embryos were removed from their droplet in a minimal amount of medium, using a micropipette under direct microscopic observation, and subjected to a live fluorescent staining, which is described below. The remaining medium was withdrawn from each droplet and collected separately in LoBind Eppendorf tubes (Eppendorf AG, Hamburg, Germany), labeled and stored at −80 °C until assayed. A corresponding volume of the incubated blank SOF/PVP/ITS medium was stored under similar conditions. 

#### 4.2.2. Determination of the Ratio of Membrane-Damaged Cells

At day 8 post-fertilization, 606 embryos of the SOF/PVP/ITS group underwent a live Hoechst/propidium iodide staining, without fixation or permeabilization treatment. In brief, individual embryos were exposed to 18.6 µg/mL propidium iodide in PBS with 0.5% BSA (PBS/BSA), for 5 min at room temperature. After a wash-step in PBS/BSA at room temperature, the embryos were transferred to a 10 μg/mL bisbenzimide (Hoechst 33342) solution in PBS/BSA, for 10 min at room temperature. Subsequently, embryos were washed (PBS/BSA), mounted individually on glass slides and stored at 4 °C. The next day, embryos were examined under an epifluorescence microscope (Leica DMR) using an A513804 filter cube (Excitation filter: BP 340-380; Dichromatic mirror: 400; Suppression filter: LP 425), allowing for the visualization of both Hoechst and propidium iodide at the same time. In this way, the TCN and the number of MDCs could be determined since propidium iodide cannot enter cells with an intact cell membrane as opposed to Hoechst. Hence, MDCs will stain pink (combination of Hoechst and propidium iodide), whilst membrane-intact cells stain blue (only Hoechst). Consequently, the MDC ratio could be calculated for each embryo as the ratio of the number of MDCs, divided by the TCN. Based on these results, the embryos were assigned a label as ‘excellent’, ‘good’ or ‘poor’ quality, with excellent-quality embryos showing normal progress to the blastocyst stage (at least 64 cells at day 8 post-fertilization) and no MDCs; good-quality embryos showing similar development with a limited number of MDCs (0.1 to 5%); and poor-quality embryos showing arrested/delayed development (less than 64 cells at day 8 post-fertilization) and having at least 90% MDCs.

#### 4.2.3. Protein Precipitation and in-Solution Trypsin Digest

After fluorescent staining, embryo-conditioned medium of the 90 good-quality embryos was pooled in order to obtain sufficient protein concentration for tandem MS analysis. Similarly, the embryo-conditioned medium of 90 randomly selected poor-quality embryos was pooled, and also the volume of blank medium corresponding to the volume of 90 droplets was pooled. These three pools of media were analyzed in MS run 1. Subsequently, embryo-conditioned media of the 26 excellent-quality embryos, 26 randomly selected poor-quality embryos, or blank medium corresponding to the volume of 26 droplets were pooled and analyzed in MS run 2. The proteins in the six pools were precipitated by adding nine volumes of ice-cold acetone and stored overnight at −20 °C. Afterward, the pools were centrifuged at 20,800 g for 10 min at 4 °C and the supernatant was discarded. The pellet, which contained the precipitated peptides, was then dried in a CentriVap Cold trap (Labconco, Kansas City, MO, USA) and solubilized in 20 µL 0.5 M triethylammonium bicarbonate (TEABC), reduced with 2 μL 10 mM dithiothreitol and incubated at 60 °C for 1h. After alkylation with 1 μL 200 mM S-methyl methane-thiosulfate (Fluka) for 10 min at room temperature, the six pools were incubated overnight at 37 °C, with trypsin (5 μg trypsin/10 μL TEABC), 1mM CaCl_2_ and 5% acetonitrile (ACN), dried in the CentriVap Cold Trap and stored at −20 °C. All steps in this section were performed using Protein LoBind^®^ Eppendorf tubes.

#### 4.2.4. Protein Screening by Mass Spectrometric Analysis

The dried peptides of the six pools were dissolved in 0.1% formic acid (FA) and separated by a 45 min gradient on reversed-phase nano-high-performance liquid chromatography (5 cm PepMap C18 analytical column, Dionex) at 60 °C, using a linear gradient of H2O/ACN (97:3, 0.1% FA) to H2O/ACN (20:80, 0.1% FA) at 300 nL/minute (Dionex U3000 Nano LC system). Peptides were analyzed by TripleTOF 5600 (Sciex, Framingham, MA, USA) in a data-dependent mode, with dynamic accumulation automatically switching between MS (400 *m*/*z*–1250 *m*/*z*; accumulation time 0.25 s) and MS/MS (65–2000 *m*/*z*; accumulation time 0.20 s) on the 20 most abundant precursors when a threshold of 50 cps was exceeded. These ions were excluded for further analysis for 4 s. Wiff files were converted to mgf format with Peakview 2.1 and data were searched against a bovine database containing reviewed UniProt accessions only (downloaded 3 August 2018), supplemented with the common Repository of Adventitious Proteins, cRAP (CrAP database, www.thegpm.org/crap/ (accessed on 3 August 2018)), using Mascot Daemon version 2.4 (Matrix Science, London, UK). Peptide mass tolerance was set at 15 ppm and fragment mass tolerance at 0.3 Da. No missed cleavages were allowed, identity threshold for peptides was set at *p* < 0.01 and a minimum of two sequences per protein was requested. 

The data were annotated using Mascot server 2.5 (Matrix Science) by searching against the UniProt Reviewed Bovine database (downloaded August 2018, 6889 sequences) supplemented with common lab contaminants (www.thegpm.org/crap (accessed on 3 August 2018)), with the following parameters: enzyme trypsin with maximum two missed cleavages, fixed modification methylthio (C), variable modifications deamidation (NQ) and oxidation (M), 15 ppm precursor mass tolerance, 0.3 Da MSMS tolerance. The ID threshold was set to an expectancy value of 0.01.

### 4.3. Confirmation of the Embryotrophic Effect of Cathepsin-L

The paracrine effect of cathepsin-L was further explored in in vitro embryo production experiments. To do so, 100 ng/mL of active human cathepsin-L (Abcam, Cambridge, UK) was supplemented to the embryo culture medium. The optimal concentration of cathepsin-L was determined in preliminary dose–response experiments (supplementary data). Cathepsin-L was stored at 4 °C, according to the manufacturer’s instructions.

#### 4.3.1. Supplementation of Cathepsin-L to Individual Culture Conditions

Immature bovine oocytes (n = 1.767, over six replicates) were harvested from slaughterhouse ovaries for in vitro maturation and fertilization, as previously described. Fertilized zygotes were cultured individually (one presumed zygote per 20 µL droplet) in cathepsin-L-supplemented medium (SOF/BSA/ITS + cathepsin-L 100 ng/mL; n = 595) or in control medium (SOF/BSA/ITS; n = 612). Since previous studies confirmed that culturing embryos individually might jeopardize embryonic development as opposed to group culture [7], an extra control group (25 presumed zygotes per 50 µL droplets of SOF/BSA/ITS medium) was included (n = 560). All droplets were covered with paraffin oil. All embryos were cultured at 38.5 °C in 5% CO_2_, 5% O_2_ and 90% N_2_, until 8 days post-fertilization.

#### 4.3.2. Assessment of Embryo Development

At 45 h post-fertilization (hpf), the embryo cleavage rate was evaluated as the percentage of presumed oocytes that cleaved. The kinetics of cleavage was assessed and embryos that proceeded to the third cleavage division (5–8 cells) at 45 hpf were classified as ‘fast cleavers’ [87], calculated as the number of zygotes cleaved into > 4 cells, relative to the total number of cleaved zygotes. Blastocyst rates were evaluated at days 7 and 8 post-fertilization as the number of blastocysts relative to the total number of fertilized oocytes. The hatching ratio was calculated at day 8 as the number of hatching or hatched blastocysts, relative to the total amount of blastocysts.

#### 4.3.3. Differential Apoptotic Staining 

**Figure 4:**
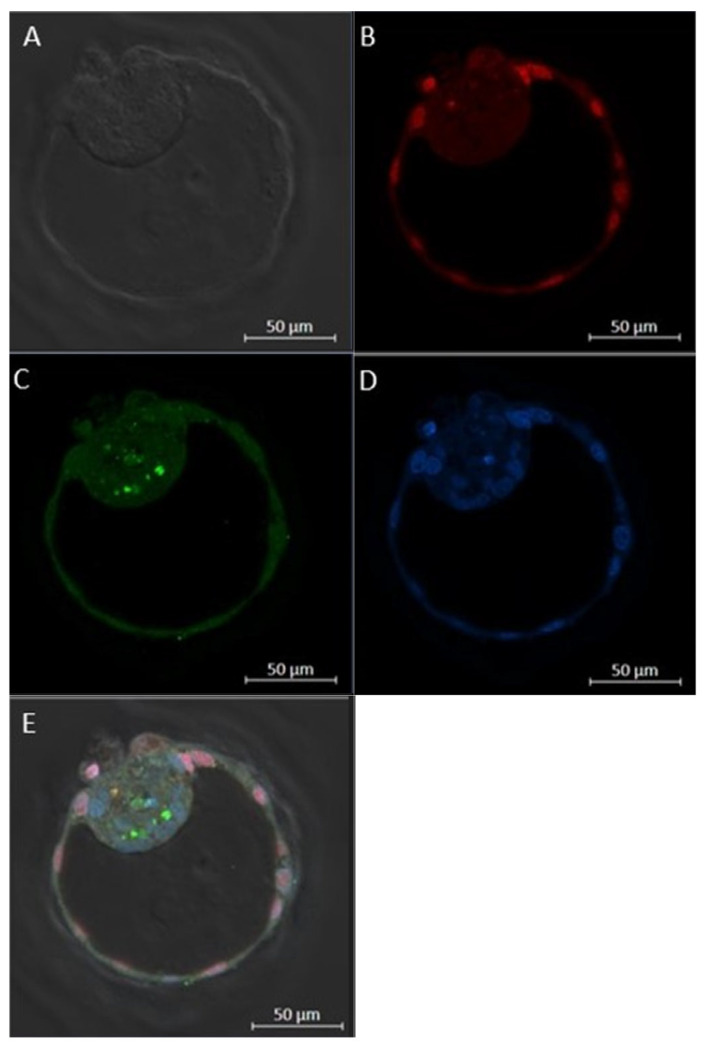
Brightfield (**A**) and fluorescent (**B**–**E**) images of differential apoptotic staining in a bovine blastocyst. At 8 days post-fertilization, bovine blastocysts were fixed and immuno-stained for CDX2 (red), (**B**) and active caspase-3 (green), (**C**). Similarly, Hoechst staining was performed to stain all nuclei (blue), (**D**). The merged image (**E**) shows the distribution of the different cell types in the blastocyst. The image was acquired by confocal microscopy using a 20x objective and represents a hatching blastocyst derived from the control group.

Blastocysts obtained from experiment 2.3.1 (control group culture: n = 38; control individual culture: n = 41; cathepsin-L individual culture: n = 32) were collected at day 8 post-fertilization and fixed in 2% paraformaldehyde (*w*/*v*) for 20 min. Subsequently, blastocysts were subjected to fluorescent differential apoptotic staining to assess the quality of their morphological development [88]. This method combines double-immunofluorescent staining against CDX2 (i.e., a transcription factor that is only expressed in trophoblast cells) and active caspase-3 (i.e., a protein involved in the apoptosis pathway) with Hoechst staining (stains nuclei of all cells). Hence, simultaneous evaluation of three important parameters of embryo quality was performed: (1) TCN, (2) inner cell mass ratio, which is the ratio of inner cell mass cells (trophoblast cell number subtracted from TCN) to the TCN, and (3) the apoptotic cell ratio, calculated as the number of apoptotic cells, divided by the TCN (Figure 4).

#### 4.3.4. Statistical Analysis

All analyses were conducted in R (version 4.2.1) and RStudio (1 July 2022 Build 554). Data on embryo development (cleavage, fast-cleavers, blastocyst, and hatching rates) were analyzed using generalized mixed-effects models fit by maximum likelihood. The treatment group was fitted as a fixed effect and the replicate was set as random. Differences among groups were assessed by the post hoc Tukey test.

The distribution of embryo quality data (resulting from differential apoptotic staining: TCN, TE, ICM, ICR, AC and ACR) were evaluated by the Shapiro–Wilk test (*p* < 0.05) and Q–Q plots. When data were not normally distributed, a log-10 transformation was performed. Normally distributed variables were analyzed by a one-way analysis of variance (ANOVA) and by the post hoc Tukey test. The homogeneity assumption required by ANOVA was evaluated using a Levene’s test. When the assumptions for ANOVA were not met, Kruskal–Wallis and post hoc Mann–Whitney U tests were performed (AC and ACR). The significance level was set at *p*-value < 0.05.

## 5. Conclusions

In this study, cathepsin-L was identified in the secretome of bovine embryos of excellent and good quality. The embryotrophic properties confirmed with cathepsin-L supplementation to the culture medium of individually cultured bovine embryos were (1) progressing blastulation, (2) stimulating blastocyst hatching and (3) improving blastocyst quality in terms of lower apoptotic cell ratio compared to individual culture in the absence of cathepsin-L. This research provides considerable resources for further studies on inter-embryo communications and explores the role of cathepsin-L as an embryotrophic signaling factor. The combined knowledge acquired through proteomics, and in vitro embryo culture will be a helpful tool to further optimize in vitro embryo culture conditions, both for human and bovine embryos.

In conclusion, cathepsin-L is actively secreted by bovine embryos of excellent and good quality that are in-vitro-produced, and the addition of this protein to the culture medium of individually cultured embryos confirmed its critical role in preimplantation embryo development.

## Figures and Tables

**Table 1:** Development of embryos cultured in cathepsin-L-supplemented medium or in the control medium.

Medium	Culture System	Cleavage (%) ^A^	Fast Cleavers (%) ^B^	Blastocyst (%) ^A^		Hatching (%) ^C^
				Day 7	Day 8	
Control	Group	83.8 ± 1.62	21.9 ± 3.06 ^a^	39.5 ± 2.07 ^a^	48.0 ± 2.11 ^a^	17.1 ± 1.81 ^a^
Control	Individual	80.4 ± 1.68	12.2 ± 2.10 ^b^	20.9 ± 1.64 ^b^	36.1 ± 1.94 ^b^	7.67 ± 1.81 ^b^
Cathepsin-L	Individual	81.9 ± 1.65	13.7 ± 2.28 ^b^	27.6 ± 1.83 ^c^	38.3 ± 1.99 ^b^	19.9 ± 2.67 ^a^

Data are represented as the least-square mean ± SE. Within each column, values that differ significantly are indicated by different superscripts (*p* < 0.05). ^A^ Cleavage and blastocysts rates are calculated as the number of cleaved zygote resp. blastocysts, divided by the total number of used presumed zygotes in the corresponding treatment group (n = 560, 612, and 595 for the control group, control individual and cathepsin-L, respectively). ^B^ The proportion of fast cleavers is shown as the percentage of zygotes that cleaved into 5 or 8 cells at 45 h post-fertilization compared to the total number of cleaved zygotes in the corresponding treatment group (n = 473, 492 and 487 for the control group, control individual and cathepsin-L, respectively). ^C^ The hatching rate was calculated as the percentage of hatching or hatched blastocysts compared to the total number of blastocysts at day 8 post-fertilization in the corresponding treatment group (n = 269, 221 and 231 for the control group, control individual and cathepsin-L, respectively).

**Table 2:** Results of embryo quality assessed by differential apoptotic staining methods.

Medium	Culture System	N° of Blastocysts	Cell Number				ICR	ACR ^A^
			TCN	ICM	TE	AC ^A^		
Control	Group	38	115.9 ± 4.55	36.2 ± 2.91	79.9 ± 3.10	6.0 ± 4.00 ^a^	30.3 ± 1.66	4.8 ± 3.90 ^a^
Control	Individual	41	117.0 ± 3.37	35.7 ± 2.24	81.5 ± 2.66	5.0 ± 4.00 ^a^	30.0 ± 1.51	4.4 ± 3.32 ^a^
Cathepsin-L	Individual	32	116.8 ± 4.30	35.4 ± 2.90	81.6 ± 4.22	4.0 ± 2.00 ^b^	30.4 ± 2.28	3.1 ± 2.59 ^b^

Data are represented as the least-square mean ± SE. ^A^AC and ACR data were not normally distributed and are therefore represented as the median ± IQR (IQR = Q3 − Q1). Within each column, values that differ significantly are indicated by different superscripts (*p* < 0.05). Used abbreviations: TCN: total cell number; ICM: inner cell mass; TE: trophoblast cells; AC: apoptotic cells; ICR: inner cell mass ratio; ACR: apoptotic cell ratio.

## Data Availability

Data are contained within the article or Supplementary Materials.

## Supplementary Materials

The following supporting information can be downloaded at: https://www.mdpi.com/article/10.3390/ijms24076563/s1.

## Abbreviations

ACN, acetonitrile; BSA, bovine serum albumin; FA, formic acid; hpf, hours post-fertilization; MDC, membrane-damaged cells; MS, mass spectrometry; PBS, phosphate-buffered saline; PVP, polyvinylpyrrolidone; TALP, Tyrode’s albumin lactate pyruvate; TCM, tissue culture medium; TCN, total cell number; TEABC, triethylammonium bicarbonate.
