# Supplementary material for: Cathepsin-L Secreted by High-Quality Bovine Embryos Exerts an Embryotrophic Effect In Vitro
Source: Int J Mol Sci. 2023 Mar 31;24(7):6563. doi: 10.3390/ijms24076563 (PMC10094850; doi:10.3390/ijms24076563)
Supplement: Supplementary file 1 [file ijms-24-06563-s001.zip › ijms-2288966-supplementary.pdf]

THE EMBRYOTROPHIC EFFECT OF CATHEPSIN-L *IN VITRO*: SUPPLEMENTARY DATA

Supplementary Table S1: Proteins identified in a pool of medium conditioned by 90 embryos of good quality (TCN > 64 cells; MDC-ratio = 0.1 - 5%) and 90 embryos of poor quality (TCN < 64 cells; MDC-ratio > 90%) and not identified in the blank.

	Accession	Score[a]	# Peptides[b] (p < 0.01)	# Sequences (p < 0.01)	Description
**'Good' quality embryos: TCN > 64 cells; MDC-ratio = 0.1 - 5 %**					
1	CATL1_BOVIN	212	2	2	Cathepsin L1
2	VTDB_BOVIN	191	3	3	Vitamin D-binding protein
3	TTHY_BOVIN	153	2	2	Transthyretin OS=Bos taurus
4	A1AG_BOVIN	81	1	1	Alpha-1-acid glycoprotein
5	MVP_BOVIN	66	1	1	Major vault protein
6	PRDX1_BOVIN	45	1	1	Peroxiredoxin-1
7	SPRC_BOVIN	44	1	1	SPARC
8	FETA_BOVIN	30	1	1	Alpha-fetoprotein
**'Poor' quality embryos: TCN < 64 cells; MDC-ratio > 90 %**					
1	MVP_BOVIN	1151	38	31	c
2	TTHY_BOVIN	739	15	7	Transthyretin
3	PEBP1_BOVIN	504	10	7	Phosphatidylethanolamine-binding protein 1
4	VTDB_BOVIN	346	7	7	Vitamin D-binding protein
5	A1AG_BOVIN	333	10	9	Alpha-1-acid glycoprotein
6	ACTB_BOVIN	278	8	7	Actin, cytoplasmic 1
7	PRDX2_BOVIN	256	9	7	Peroxiredoxin-2
8	FABPH_BOVIN	221	4	3	Fatty acid-binding protein, heart
9	INPP_BOVIN	217	5	4	Inositol polyphosphate 1-phosphatase
10	PRDX1_BOVIN	215	9	7	Peroxiredoxin-1
11	LYSM_BOVIN	191	3	1	Lysozyme C, milk isozyme
12	SODC_BOVIN	185	5	4	Superoxide dismutase [Cu-Zn]
13	SPA31_BOVIN	158	6	4	Serpin A3-1
14	ACTC_BOVIN	153	6	5	Actin, alpha cardiac muscle 1
15	CASA1_BOVIN	152	5	4	Alpha-S1-casein
16	LDHA_BOVIN	149	4	3	L-lactate dehydrogenase A chain
17	ACTA_BOVIN	147	5	4	Actin, aortic smooth muscle
18	UCHL1_BOVIN	146	3	3	Ubiquitin carboxyl-terminal hydrolase isozyme L1
19	G6PI_BOVIN	140	2	1	Glucose-6-phosphate isomerase
20	SPA35_BOVIN	127	5	3	Serpin A3-5
21	LDHB_BOVIN	127	4	3	L-lactate dehydrogenase B chain
22	NDKB_BOVIN	111	6	4	Nucleoside diphosphate kinase B
23	PARK7_BOVIN	111	2	2	Protein/nucleic acid deglycase DJ-1
24	LACB_BOVIN	110	2	2	Beta-lactoglobulin
25	SPA33_BOVIN	108	3	2	Serpin A3-3
26	AK1A1_BOVIN	108	3	3	Alcohol dehydrogenase [NADP(+)]
27	PDIA3_BOVIN	105	5	5	Protein disulfide-isomerase A3
28	CH10_BOVIN	102	2	1	10kDa heat shock protein, mitochondrial
29	NDKA1_BOVIN	99	5	3	Nucleoside diphosphate kinase A 1

30	BIP_BOVIN	98	3	3	Endoplasmic reticulum chaperone BiP
31	PRDX4_BOVIN	96	5	3	Peroxiredoxin-4
32	TYB4_BOVIN	93	1	1	Thymosin beta-4
33	ALDR_BOVIN	84	3	3	Aldose reductase
34	CALM_BOVIN	76	1	1	Calmodulin
35	PDIA1_BOVIN	74	1	1	Protein disulfide-isomerase
36	CASA2_BOVIN	72	2	2	Alpha-S2-casein
37	ARGI1_BOVIN	72	2	2	Arginase-1
38	ADA_BOVIN	72	2	2	Adenosine deaminase
39	SBP1_BOVIN	70	1	1	Selenium-binding protein 1
40	FKB1A_BOVIN	70	1	1	Peptidyl-prolyl cis-trans isomerase FKBP1A
41	RS27A_BOVIN	69	1	1	Ubiquitin-40S ribosomal protein S27a
42	FABP5_BOVIN	67	3	3	Fatty acid-binding protein, epidermal
43	IDHC_BOVIN	60	2	2	Isocitrate dehydrogenase [NADP] cytoplasmic
44	H2B1N_BOVIN	57	1	1	Histone H2B type 1-N
45	PRDX3_BOVIN	55	1	1	Thioredoxin-dependent peroxide reductase, mitochondrial
46	GELS_BOVIN	54	1	1	Gelsolin
47	ZP4_BOVIN	54	1	1	Zona pellucida sperm-binding protein 4
48	PHS_BOVIN	54	2	2	Pterin-4-alpha-carbinolamine dehydratase
49	HBA_BOVIN	54	2	2	Hemoglobin subunit alpha
50	TPM1_BOVIN	53	1	1	Tropomyosin alpha-1 chain
51	COF1_BOVIN	53	1	1	Cofilin-1
52	MIF_BOVIN	53	1	1	Macrophage migration inhibitory factor
53	VNN1_BOVIN	52	1	1	Pantetheinase
54	UCHL3_BOVIN	50	1	1	Ubiquitin carboxyl-terminal hydrolase isozyme L3
55	SPA38_BOVIN	49	1	1	Serpin A3-8
56	FETA_BOVIN	46	4	1	Alpha-fetoprotein
57	SPA37_BOVIN	46	2	1	Serpin A3-7
58	CDK5_BOVIN	46	1	1	Cyclin-dependent-like kinase 5
59	G3P_BOVIN	45	1	1	Glyceraldehyde-3-phosphate dehydrogenase
60	CALU_BOVIN	45	2	2	Calumenin
61	HSP7C_BOVIN	43	1	1	Heat shock cognate 71 kDa protein
62	AATC_BOVIN	42	1	1	Aspartate aminotransferase, cytoplasmic
63	HPT_BOVIN	41	1	1	Haptoglobin
64	MDHC_BOVIN	38	1	1	Malate dehydrogenase, cytoplasmic
65	AOCX_BOVIN	38	1	1	Primary amine oxidase, liver isozyme
66	PPIB_BOVIN	36	1	1	Peptidyl-prolyl cis-trans isomerase B
67	TYB10_BOVIN	35	1	1	Thymosin beta-10
68	CALR_BOVIN	35	2	2	Calreticulin
69	IMPA1_BOVIN	34	1	1	Inositol monophosphatase 1
70	CASK_BOVIN	34	1	1	Kappa-casein
71	RHG29_BOVIN	33	1	1	Rho GTPase-activating protein 29
72	ASGL1_BOVIN	32	1	1	Isoaspartyl peptidase/L-asparaginase
73	HSPB1_BOVIN	32	1	1	Heat shock protein beta-1
74	FIBB_BOVIN	32	1	1	Fibrinogen beta chain
75	DSG1_BOVIN	32	1	1	Desmoglein-1
76	H2A1_BOVIN	32	1	1	Histone H2A type 1

77	EZRI_BOVIN	30	1	1	Ezrin
78	CFAB_BOVIN	30	1	1	Complement factor B
79	DNPEP_BOVIN	29	1	1	Aspartyl aminopeptidase
80	PROF1_BOVIN	28	1	1	Profilin-1
81	ARC1B_BOVIN	26	1	1	Actin-related protein 2/3 complex subunit 1B
82	NNRE_BOVIN	26	1	1	NAD(P)H-hydrate epimerase
83	CPT1B_BOVIN	26	1	1	Carnitine O-palmitoyltransferase 1, muscle isoform
84	AATM_BOVIN	26	1	1	Aspartate aminotransferase, mitochondrial
85	UACA_BOVIN	26	1	1	Uveal autoantigen with coiled-coil domains and ankyrin repeats protein

[a] The score of a peptide is a measure for the quality of the spectrum obtained after MSMS.

[b] The number of identified peptides is mentioned as a rough estimate of the abundance of this protein in the sample.

Supplementary Table S2: Proteins identified in a pool of medium conditioned by 26 embryos of 'excellent' quality (TCN > 64 cells; MDC-ratio = 0%) and 26 embryos of 'poor' quality (TCN < 64 cells; MDC-ratio > 90%) and not identified in the blank.

	Accession	Score[a]	# Peptides[b] (p < 0.01)	# Sequences (p < 0.01)	Description
**'Excellent' quality embryos: TCN > 64 cells; MDC-ratio = 0 %**					
1	A1AG_BOVIN	602	23	7	c
2	CASA1_BOVIN	412	15	4	Alpha-S1-casein
3	VTDB_BOVIN	337	15	6	Vitamin D-binding protein
4	SPA31_BOVIN	246	10	5	Serpin A3-1
5	TTHY_BOVIN	227	7	2	Transthyretin
6	SPA33_BOVIN	171	6	2	Serpin A3-3
7	ZA2G_BOVIN	106	6	5	Zinc-alpha-2-glycoprotein
8	AOCX_BOVIN	94	3	3	Primary amine oxidase, liver isozyme
9	MVP_BOVIN	91	4	4	Major vault protein
10	AOCY_BOVIN	86	2	2	Primary amine oxidase, lung isozyme
11	VNN1_BOVIN	81	3	3	Pantetheinase
12	ACTB_BOVIN	79	1	1	Actin, cytoplasmic 1
13	H2A1_BOVIN	78	1	1	Histone H2A type 1
14	RS27A_BOVIN	75	2	1	Ubiquitin-40S ribosomal protein S27a
15	LACB_BOVIN	71	3	2	Beta-lactoglobulin
16	SPA38_BOVIN	68	2	1	Serpin A3-8
17	K1C20_BOVIN	66	1	1	Keratin, type I cytoskeletal 20
18	ALDR_BOVIN	52	1	1	Aldose reductase
19	TPIS_BOVIN	50	1	1	Triosephosphate isomerase
20	FETA_BOVIN	49	10	1	Alpha-fetoprotein
21	FETUA_BOVIN	45	1	1	Alpha-2-HS-glycoprotein
22	PRDX1_BOVIN	44	1	1	Peroxiredoxin-1
23	FABPH_BOVIN	44	1	1	Fatty acid-binding protein, heart
24	AOC3_BOVIN	41	1	1	Membrane primary amine oxidase
25	AMBP_BOVIN	40	1	1	Protein AMBP
26	PEBP1_BOVIN	38	2	2	Phosphatidylethanolamine-binding protein 1
27	KNG1_BOVIN	38	2	2	Kininogen-1
28	ZP4_BOVIN	38	1	1	Zona pellucida sperm-binding protein 4
29	CA226_BOVIN	36	1	1	Uncharacterized protein C1orf226 homolog
30	CASA2_BOVIN	34	3	2	Alpha-S2-casein
31	PARK7_BOVIN	33	1	1	Protein/nucleic acid deglycase DJ-1
32	PRDX6_BOVIN	32	1	1	Peroxiredoxin-6
33	ANT3_BOVIN	31	1	1	Antithrombin-III
34	DSC1_BOVIN	31	1	1	Desmocollin-1
35	CAB39_BOVIN	31	1	1	Calcium-binding protein 39
36	K2C74_BOVIN	29	1	1	Keratin, type II cytoskeletal 74
37	CATL1_BOVIN	26	2	2	Cathepsin L1
38	NDKB_BOVIN	26	1	1	Nucleoside diphosphate kinase B
39	INPP_BOVIN	26	1	1	Inositol polyphosphate 1-phosphatase
40	SODC_BOVIN	25	1	1	Superoxide dismutase [Cu-Zn]
41	ORC4_BOVIN	24	1	1	Origin recognition complex subunit 4

42	LYSM_BOVIN	23	1	1	Lysozyme C, milk isozyme
43	REXO5_BOVIN	22	1	1	RNA exonuclease 5
**'Poor' quality embryos: TCN < 64 cells; MDC-ratio > 90 %**					
1	MVP_BOVIN	1429	52	31	Major vault protein
2	A1AG_BOVIN	1015	29	9	Alpha-1-acid glycoprotein
3	TTHY_BOVIN	956	25	7	Transthyretin
4	PEBP1_BOVIN	825	25	9	Phosphatidylethanolamine-binding protein 1
5	VTDB_BOVIN	513	17	8	Vitamin D-binding protein
6	NDKB_BOVIN	493	11	4	Nucleoside diphosphate kinase B
7	NDKA1_BOVIN	441	8	3	Nucleoside diphosphate kinase A 1
8	SODC_BOVIN	411	7	3	Superoxide dismutase [Cu-Zn]
9	G6PI_BOVIN	401	11	9	Glucose-6-phosphate isomerase
10	INPP_BOVIN	316	12	7	Inositol polyphosphate 1-phosphatase
11	SPA31_BOVIN	301	10	8	Serpin A3-1
12	PRDX6_BOVIN	290	10	9	Peroxiredoxin-6
13	SPA32_BOVIN	280	9	7	Serpin A3-2
14	PDIA3_BOVIN	264	10	10	Protein disulfide-isomerase A3
15	KCRU_BOVIN	260	7	5	Creatine kinase U-type, mitochondrial
16	PRDX2_BOVIN	244	9	8	Peroxiredoxin-2
17	FABPH_BOVIN	242	8	6	Fatty acid-binding protein, heart
18	CALR_BOVIN	234	6	4	Calreticulin
19	ACTB_BOVIN	232	7	5	Actin, cytoplasmic 1
20	ADA_BOVIN	226	7	7	Adenosine deaminase
21	ACTC_BOVIN	215	6	4	Actin, alpha cardiac muscle 1
22	BIP_BOVIN	211	6	6	Endoplasmic reticulum chaperone BiP
23	LYSM_BOVIN	210	6	1	Lysozyme C, milk isozyme
24	SPA33_BOVIN	205	5	4	Serpin A3-3
25	SPA35_BOVIN	204	4	3	Serpin A3-5
26	ENOA_BOVIN	204	3	2	Alpha-enolase
27	LDHA_BOVIN	192	7	5	L-lactate dehydrogenase A chain
28	IDHC_BOVIN	183	8	8	Isocitrate dehydrogenase [NADP] cytoplasmic
29	LACB_BOVIN	181	3	3	Beta-lactoglobulin
30	DNPEP_BOVIN	178	5	5	Aspartyl aminopeptidase
31	PARK7_BOVIN	178	6	5	Protein/nucleic acid deglycase DJ-1
32	ENOB_BOVIN	175	2	1	Beta-enolase
33	CASA1_BOVIN	173	6	3	Alpha-S1-casein
34	TYB4_BOVIN	172	4	2	Thymosin beta-4
35	ACTA_BOVIN	171	5	3	Actin, aortic smooth muscle
36	SODM_BOVIN	159	6	2	Superoxide dismutase [Mn], mitochondrial
37	CALU_BOVIN	147	5	4	Calumenin
38	CALM_BOVIN	146	4	3	Calmodulin
39	AK1A1_BOVIN	138	6	6	Alcohol dehydrogenase [NADP(+)]
40	AATM_BOVIN	135	5	5	Aspartate aminotransferase, mitochondrial
41	PDIA1_BOVIN	130	5	4	Protein disulfide-isomerase
42	ALDR_BOVIN	126	3	3	Aldose reductase
43	CH10_BOVIN	123	3	2	10 kDa heat shock protein, mitochondrial
44	PRDX1_BOVIN	120	7	6	Peroxiredoxin-1
45	UCHL1_BOVIN	116	4	4	Ubiquitin carboxyl-terminal hydrolase isozyme L1

46	ZP4_BOVIN	115	2	2	Zona pellucida sperm-binding protein 4
47	TPIS_BOVIN	110	4	3	Triosephosphate isomerase
48	ATPA_BOVIN	104	1	1	ATP synthase subunit alpha, mitochondrial
49	UCHL3_BOVIN	103	3	3	Ubiquitin carboxyl-terminal hydrolase isozyme L3
50	ARGI1_BOVIN	101	3	3	Arginase-1
51	AMY1_HUMAN	99	3	2	Alpha-amylase 1
52	FKB1A_BOVIN	98	2	2	Peptidyl-prolyl cis-trans isomerase FKBP1A
53	AOCX_BOVIN	94	4	4	Primary amine oxidase, liver isozyme
54	SBP1_BOVIN	91	4	4	Selenium-binding protein 1
55	PROF1_BOVIN	87	2	2	Profilin-1
56	LDHB_BOVIN	85	4	4	L-lactate dehydrogenase B chain
57	SPA38_BOVIN	84	2	2	Serpin A3-8
58	PGAM1_BOVIN	80	3	3	Phosphoglycerate mutase 1
59	AOC3_BOVIN	79	2	2	Membrane primary amine oxidase
60	SUMO2_BOVIN	78	1	1	Small ubiquitin-related modifier 2
61	H2A1_BOVIN	77	1	1	Histone H2A type 1
62	ORN_BOVIN	75	2	2	Oligoribonuclease, mitochondrial
63	QOR_BOVIN	74	1	1	Zeta-crystallin
64	SH3L3_BOVIN	74	2	2	SH3 domain-binding glutamic acid-rich-like protein 3
65	NASP_BOVIN	70	2	2	Nuclear autoantigenic sperm protein
66	ASGL1_BOVIN	70	4	4	Isoaspartyl peptidase/L-asparaginase
67	LDH6B_BOVIN	67	2	1	L-lactate dehydrogenase A-like 6B
68	HPT_BOVIN	67	2	2	Haptoglobin
69	K1C20_BOVIN	66	1	1	Keratin, type I cytoskeletal 20
70	CATA_BOVIN	65	1	1	Catalase
71	G3P_BOVIN	63	2	2	Glyceraldehyde-3-phosphate dehydrogenase
72	MYDGF_BOVIN	63	2	2	Myeloid-derived growth factor
73	PRDX4_BOVIN	62	4	3	Peroxiredoxin-4
74	STMN1_BOVIN	60	2	2	Stathmin
75	AOCY_BOVIN	57	1	1	Primary amine oxidase, lung isozyme
76	PRDX3_BOVIN	57	3	2	Thioredoxin-dependent peroxide reductase, mitochondrial
77	CFAB_BOVIN	57	1	1	Complement factor B
78	FETA_BOVIN	56	12	1	Alpha-fetoprotein
79	MIF_BOVIN	55	2	1	Macrophage migration inhibitory factor
80	GGCT_BOVIN	54	1	1	Gamma-glutamylcyclotransferase
81	ACPM_BOVIN	54	1	1	Acyl carrier protein, mitochondrial
82	CASK_BOVIN	53	1	1	Kappa-casein
83	HBA_BOVIN	53	1	1	Hemoglobin subunit alpha
84	ANXA2_BOVIN	52	1	1	Annexin A2
85	CLUS_BOVIN	52	2	2	Clusterin
86	PPIB_BOVIN	52	4	3	Peptidyl-prolyl cis-trans isomerase B
87	DSG1_BOVIN	52	2	2	Desmoglein-1
88	1433Z_BOVIN	51	1	1	14-3-3 protein zeta/delta SV=1
89	FETUA_BOVIN	51	1	1	Alpha-2-HS-glycoprotein
90	IMPA1_BOVIN	51	2	2	Inositol monophosphatase 1
91	HSP7C_BOVIN	50	1	1	Heat shock cognate 71 kDa protein
92	FPPS_BOVIN	50	2	2	Farnesyl pyrophosphate synthase
93	ACTN1_BOVIN	50	1	1	Alpha-actinin-1

94	ATOX1_BOVIN	48	1	1	Copper transport protein ATOX1
95	NALP5_BOVIN	48	1	1	NACHT, LRR and PYD domains-containing protein 5
96	THOP1_BOVIN	48	2	2	Thimet oligopeptidase
97	KNG1_BOVIN	47	1	1	Kininogen-1
98	A1AT_BOVIN	47	1	1	Alpha-1-antiproteinase
99	FHIT_BOVIN	44	2	2	Bis(5~-adenosyl)-triphosphatase
100	ZA2G_BOVIN	44	4	4	Zinc-alpha-2-glycoprotein
101	ODO2_BOVIN	42	1	1	Dihydrolipoyllysine-residue succinyl-transferase component of 2-oxoglutarate dehydrogenase complex, mitochondrial
102	TPM1_BOVIN	42	1	1	Tropomyosin alpha-1 chain
103	HSPB1_BOVIN	41	1	1	Heat shock protein beta-1
104	NTF2_BOVIN	41	1	1	Nuclear transport factor 2
105	ANT3_BOVIN	41	1	1	Antithrombin-III
106	1433E_BOVIN	40	1	1	14-3-3 protein epsilon
107	AATC_BOVIN	40	2	2	Aspartate aminotransferase, cytoplasmic
108	OOEP_BOVIN	38	1	1	Oocyte-expressed protein homolog
109	PDIA4_BOVIN	38	1	1	Protein disulfide-isomerase A4
110	LSM3_BOVIN	38	1	1	U6 snRNA-associated Sm-like protein LSm3
111	EF1A1_BOVIN	38	1	1	Elongation factor 1-alpha 1
112	MDHC_BOVIN	37	2	2	Malate dehydrogenase, cytoplasmic
113	GDIR1_BOVIN	35	1	1	Rho GDP-dissociation inhibitor 1
114	UGPA_BOVIN	34	1	1	UTP--glucose-1-phosphate uridylyltransferase
115	PHS_BOVIN	34	3	2	Pterin-4-alpha-carbinolamine dehydratase
116	PPIC_BOVIN	32	1	1	Peptidyl-prolyl cis-trans isomerase C
117	GNS_BOVIN	32	1	1	N-acetylglucosamine-6-sulfatase
118	GLRX1_BOVIN	31	1	1	Glutaredoxin-1
119	RS27A_BOVIN	31	1	1	Ubiquitin-40S ribosomal protein S27a
120	H2A2C_BOVIN	31	1	1	Histone H2A type 2-C
121	RHG29_BOVIN	29	1	1	Rho GTPase-activating protein 29
122	CPT1B_BOVIN	29	1	1	Carnitine O-palmitoyltransferase 1, muscle isoform
123	NNRE_BOVIN	27	1	1	NAD(P)H-hydrate epimerase
124	PTRD1_BOVIN	27	1	1	Putative peptidyl-tRNA hydrolase PTRHD1
125	FKBP2_BOVIN	26	1	1	Peptidyl-prolyl cis-trans isomerase FKBP2
126	ZP3_BOVIN	25	1	1	Zona pellucida sperm-binding protein 3
127	B2MG_BOVIN	22	1	1	Beta-2-microglobulin

[a] The score of a peptide is a measure for the quality of the spectrum obtained after MSMS.

[b] The number of identified peptides is mentioned as a rough estimate of the abundance of this protein in the sample.

Supplementary Table S3. Dose-response curve of cathepsin-L: development of embryos cultured in groups in three different concentrations of cathepsin-L (1 µg/ml, 100 ng/ml and 10 ng/ml) and in control medium.

Medium	Cleavage Rate[A]	Fast Cleavers[B]	Blastocyst Rate[A]		Hatching Rate[C]
			168 hpi	192 hpi	
Control	90.3 ± 2.37	11.2 ± 2.93 [a]	23.9 ± 3.42	36.8 ± 3.87	21.1 ± 5.40
1 µg/ml	88.2 ± 2.27	22.1 ± 3.69 [ab]	23.6 ± 2.98	37.9 ± 3.41	22.1 ± 4.73
100 ng/ml	85.1 ± 2.70	25.8 ± 4.29 [b]	31.6 ± 3.52	43.1 ± 3.75	26.7 ± 5.11
10 ng/ml	88.4 ±2.27	16.8 ± 3.24 [ab]	23.6 ± 3.01	38.2 ± 3.44	11.8 ± 3.71

Data are represented as least-square mean ± SE. Within each column, values that differ significantly are indicated by different superscripts ($p < 0.05$). Immature bovine oocytes (n = 1.202, 4 replicates) were harvested from slaughterhouse ovaries for *in vitro* maturation and fertilization, as previously described. Fertilized zygotes were cultured in groups (25 presumed zygote per 50 µl droplet) in medium supplemented with different concentrations of cathepsin-L (1 µg/ml: n = 203; 100 ng/ml, n = 174; 10 ng/ml, n = 199) or in control medium (SOF/BSA/ITS; n = 115). All droplets were covered with paraffin oil and embryos were cultured at 38.5°C in 5% CO_2, 5% O_2 and 90% N_2 until 192 hpi. [A] Cleavage and Blastocysts rates are calculated as the number of cleaved zygotes resp. blastocysts divided by the total number of presumed zygotes in the corresponding treatment group (n = 155, 203, 174 and 199 for control, 1 µg/ml, 100 ng/ml and 10 ng/ml respectively). [B] The proportion of fast cleavers is shown as the percentage of zygotes that cleaved into 5- or 8-cells at 45hpi compared to the total number of cleaved zygotes in the corresponding treatment group (n = 140, 179, 148 and 176 for control, 1 µg/ml, 100 ng/ml and 10 ng/ml respectively). [C] The hatching rate was calculated as the percentage of hatching or hatched blastocysts compared to the total number of blastocysts at 192 hp in the corresponding treatment group (n = 57, 77, 75 and 76 for control, 1 µg/ml, 100 ng/ml and 10 ng/ml respectively).
